# Role of TbFe on Perpendicular Magnetic Anisotropy and Giant Magnetoresistance Effect in [Co/Ni]_N_-Based Spin Valves

**DOI:** 10.1007/s40820-014-0009-1

**Published:** 2014-09-23

**Authors:** Minghong Tang, Zongzhi Zhang, Yanyan Zhu, Bin Ma, Qinyuan Jin

**Affiliations:** 1 Department of Optical Science and Engineering, Shanghai Ultra-Precision Optical Engineering Center, Fudan University, Shanghai, 200433 People’s Republic of China; 2 State Key Laboratory of Precision Spectroscopy and Department of Physics, East China Normal University, Shanghai, 200062 People’s Republic of China

**Keywords:** Nano magnetic films, Perpendicular magnetic anisotropy, Spin valves, TbFe

## Abstract

The exchange-coupled [Co/Ni]_N_/TbFe nano-magnetic films can display strong perpendicular magnetic anisotropy (PMA) which depends on the Tb:Fe component ratio, TbFe layer thickness and the repetition number N of [Co/Ni]_N_ multilayer. Perpendicular spin valves in the nano thickness scale, consisting of a [Co/Ni]_3_ free and a [Co/Ni]_5_/TbFe reference multilayer, show high giant magnetoresistance (GMR) signal of 6.5 % and a large switching field difference over 3 kOe. However, unexpected slanting of the free layer magnetization, accompanied by a reduced GMR ratio, was found to be caused by the presence of a thick Fe-rich or even a thin but Tb-rich TbFe layer. We attribute this phenomenon to the large magnetostriction effect of TbFe which probably induces strong stress acting on the free layer and hence reduces its interfacial PMA.

## Introduction

Giant magnetoresistive (GMR) devices consisting of two nano magnetic layers with perpendicular magnetic anisotropy (PMA) separated by a nonmagnetic spacer have attracted much interest for their potential applications in high density spin-transfer-torque magnetic random access memories (STT-MRAMs), where spin polarized current could be used to reverse the magnetization orientation [1–4]. In order to achieve high GMR signal and low switching current, various perpendicular magnetic films, such as Co (or CoFe)/Pt (or Ni, Pd) multilayers (MLs) [5–8], amorphous rare earth-transition metal alloys [9–11], or thin CoFeB [4, 12, 13] have been investigated. Among those structures, the ferromagnetic [Co/Ni]_N_ is considered as one of the most promising free or/and reference layer materials due to its relatively high spin polarization and small Gilbert damping factor [2, 14, 15]. However, although the perpendicular coercivity (*H*_c⊥_) of [Co/Ni]_N_ can be increased by tuning the structural parameters such as the repetition number N, or the layer thicknesses of seed layer and magnetic sublayers, the enhancement is very limited. In order to prevent simultaneous switching of the Co/Ni free and reference MLs, the reference layer switching field should be enlarged. An appropriate approach is to use a rare earth-transition metal (RE-TM) layer coupled with [Co/Ni]_N_ because the RE-TM alloy film in a proper composition ratio can display strong PMA and tunable net magnetization. In our previous work, we have fabricated spin valves (SVs) with a perpendicular [Co/Ni]_N_/TbCo composite reference layer structure, which displayed prominent features including significant switching field difference between the free and reference layers, stable GMR ratio, and negligible offset in the minor GMR curves [10]. In order to take full advantage of such composite reference layer structure, it is very necessary to examine the role of other kinds of RE-TM materials on the perpendicular magnetic properties and GMR signal of Co/Ni-based SVs.

In this paper, we present our study on perpendicular magnetic [Co/Ni]_N_/TbFe multilayers both in Tb-rich and Fe-rich conditions. High perpendicular coercivity *H*_c⊥_ of the composite film were obtained by adjusting the TbFe thickness and the repetition number N. Surprisingly, the application of TbFe layer in Co/Ni-based SVs shows considerable influence on the free layer PMA and GMR signal, and both of them decrease strongly with increasing TbFe thickness or Tb content, which are quite different from the experimental results for SVs containing a [Co/Ni]_N_/TbCo reference layer [10].

## Experiments

All the samples were deposited onto glass substrates at ambient temperature in a Kurt J. Lesker magnetron sputter system with a base pressure better than 1.0 × 10^−8^ Torr. Series of samples, in structures of Ta (3.0)/Tb_*x*_Fe_1-*x*_ (*t*)/Ta (3.0) and Ta (3.0)/Cu (2.0)/[Co (0.27)/Ni (0.59)]_N_/Tb_*x*_Fe_1-*x*_ (*t*)/Ta (3.0) (layer thickness in unit of nm) were firstly grown, where the bilayer number N changes from N = 2–10, and the TbFe thickness *t* is in the range of 2.0–12.0 nm. The TbFe alloy layer was fabricated by co-sputtering from pure Tb and Fe targets in an Ar pressure of 8.0 mTorr, and their relative atomic concentration *x*, varying from 18 to 32 %, was controlled by varying the sputtering power of Tb. Then, perpendicular spin valve stacks were prepared with a [Ni (0.59)/Co (0.27)]_3_ free layer and a composite [Co (0.27)/Ni (0.59)]_5_/Tb_*x*_Fe_1-*x*_ (*t*) reference layer. For those films, the deposition rates of Ta, Cu, Co, and Ni layers were fixed to be 0.44, 0.60, 0.45, and 0.59 Å/s, respectively.

The TbFe composition was determined by X-ray Photoelectron Spectroscopy (XPS). Magnetic properties were characterized by polar magneto-optical Kerr effect (MOKE) and a vibrating sample magnetometer (VSM). The GMR ratio measurement was conducted in a current-in-plane geometry with magnetic field applied out of plane. All the measurements were executed at room temperature (RT).

## Results and Discussion

**Fig. 1:**
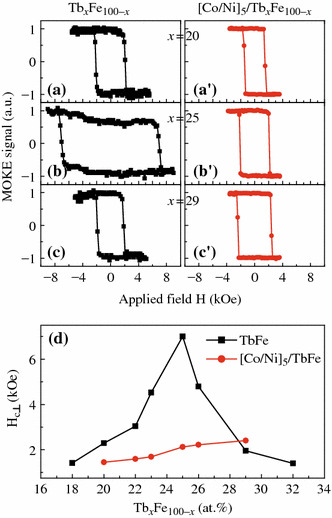
(*Color online*) Normalized polar MOKE loops for samples of Ta (3.0)/Tb_*x*_Fe_100-*x*_(6.0)/Ta(3.0) (**a**–**c**) and of Ta (3.0)/Cu (2.0)/[Co (0.27)/Ni (0.59)]_5_/Tb_*x*_Fe_100-*x*_ (6.0)/Ta (3.0) (**a**′–**c**′), in which the Tb content is 20, 25, or 29 % (atomic ratio), respectively. **d** Perpendicular coercivity *H*_c⊥_ of the above structures as a function of Tb content

Depending on the film structure and Tb content, the Tb_*x*_Fe_1-*x*_ film can display quite strong PMA, which can be employed to fix the magnetization of Co/Ni multilayers. Figure 1a–c show the normalized polar MOKE loops for Ta (3.0)/Tb_*x*_Fe_1-*x*_ (6.0)/Ta (3.0) (unit: nm) samples with different compositions of *x* = 20, 25, and 29 at%. For comparison, the polar Kerr loops for the corresponding exchange-coupled composite structure of Ta (3.0)/Cu (2.0)/[Co (0.27)/Ni (0.59)]_5_/Tb_*x*_Fe_1-*x*_ (6.0)/Ta (3) are also shown in Fig. 1a′–c′. The square-shaped loops indicate that the easy axes of all these samples are perpendicular to the film plane, but the perpendicular coercivity *H*_c⊥_ is very sensitive to the Tb content. Figure 1d plots the *H*_c⊥_ dependence on Tb content for both pure TbFe and [Co/Ni]_5_/TbFe composite films. The *H*_c⊥_ of TbFe alloy firstly increases with adding Tb content of *x*, and then decreases after reaching a maximum of 7 kOe at *x* = 25 %. Apparently, such varying behaviors of *H*_c⊥_ for the two series of samples are quite similar to the previous results reported in TbCo system [10]. It is known that the TbFe films are in a ferrimagnetic structure with Tb moments coupled antiparallel to those of the Fe. The Tb content of *x* = 25 %, at which the maximum *H*_c⊥_ occurs, corresponds to the RT compensation composition, i.e., the net magnetization of the alloy is zero. For the perpendicularly exchange-coupled [Co/Ni]_5_/TbFe composite films, there is an approximately linear relationship between Tb content and the effective *H*_c⊥_. When the Tb content *x* is lower than, or closer to the compensation composition, the *H*_c⊥_ value drops considerably compared to that of the pure TbFe samples with the same Tb content. However, for the sample with higher Tb content (*x* = 29 %), the *H*_c⊥_ is higher than the pure TbFe. It is understandable because the net magnetization of Tb-rich TbFe is dominated by Tb, which can be compensated at the interface region by the antiparallel magnetic moments of the adjacent Co/Ni. In spite of the reduced *H*_c⊥_ in the coupled [Co/Ni]_5_/TbFe structure at *x* < 29 %, the switching field is still greatly higher than that of the pure Co/Ni multilayer or Co/Ni pinned by a traditional antiferromagnetic FeMn or MnIr layer [6], which is enough to realize separate magnetization switching in [Co/Ni]_N_-based GMR structures.

**Fig. 2:**
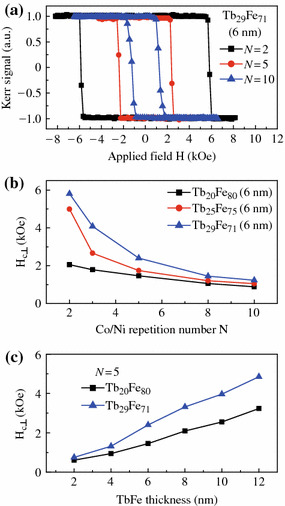
(*Color online*) **a** Polar magnetic Kerr loops of Ta (3.0)/Cu (2.0)/[Co/Ni]_N_/Tb_29_Fe_71_ (6.0)/Ta (3.0) for N = 2, 5, and 10. **b** Perpendicular coercivity *H*_c⊥_ as a function of Co/Ni repeats N for different Tb contents. **c** Perpendicular coercivity as a function of TbFe layer thickness for N = 5 case

The magnitude of the perpendicular *H*_c⊥_ for the composite structure can also be tuned in a wide range by varying the multilayer repetition number N of [Co/Ni]_N_ or the TbFe thickness. Figure 2a displays the normalized Kerr loops for samples of Ta (3.0)/Cu (2.0)/[Co (0.27)/Ni (0.59)]_N_/Tb_29_Fe_71_ (6.0)/Ta (3.0) for N = 2, 5, and 10. The definite square loops reveal strong out-of-plane magnetic anisotropy, and the single-step switching suggests that the perpendicular TbFe and [Co/Ni]_N_ layers are exchange coupled rigidly, behaving as a single layer even for N = 10. Typical exchange-coupled samples with three different TbFe contents, designated as Fe-rich (Tb_20_Fe_80_), compensation point (Tb_25_Fe_75_), and Tb-rich (Tb_29_Fe_71_), are chosen to study the relationship between the out-of-plane coercivity and the Co/Ni repetition number N. As shown in Fig. 2b, clearly, the perpendicular switching fields of the three composite structures all decrease with increasing N, reaching almost a constant value at N = 10. Such varying behavior of *H*_c⊥_ is very similar to the common hard/soft exchange-coupled systems with in-plane magnetic anisotropy [16, 17]. In addition to the repetition number N, the effective out-of-plane coercivity can also be manipulated by varying the TbFe thickness. As seen in Fig. 2c for N = 5 case, the *H*_c⊥_ value increases rapidly with TbFe layer thickness for both the Fe-rich (*x* = 20) and Tb-rich (*x* = 29) samples. The *H*_c⊥_ value of the composite film with a 12 nm thick of Tb_29_Fe_71_ is as high as 4.85 kOe, almost 6 times higher than that with a 2 nm thick of Tb_29_Fe_71_. Note that the *H*_c⊥_ rise rate of Tb-rich sample is faster than the Fe-rich, which should result from the additional magnetic moment compensation effect from the adjacent transition metal in [Co/Ni]_N_ multilayers.

**Fig. 3:**
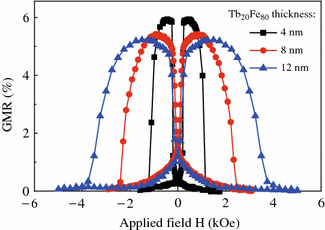
(*Color online*) The GMR curves for spin valves of Ta (3.0)/Cu (1.4)/[Ni/Co]_3_/Cu (2.2)/[Co/Ni]_5_/Tb_20_Fe_80_ (*t*_1_ = 4.0, 8.0, or 12.0 nm)/Ta (3.0)

Based on the above experimental results, we find that an enhanced and tunable switching field can be achieved by capping a thin TbFe alloy on top of [Co/Ni]_N_. Therefore, such composite structure can be applied to the SVs as reference layer to achieve large switching field differences. Figure 3 shows the thickness effect of Fe-rich Tb_20_Fe_80_ layer on the GMR transfer curves for samples of Ta (3.0)/Cu (1.4)/[Ni (0.59)/Co (0.27)]_3_/Cu (2.2)/[Co (0.27)/Ni (0.59)]_5_/Tb_20_Fe_80_ (*t*_1_)/Ta (3.0). Obviously, for the sample with a thin Tb_20_Fe_80_ layer of *t*_1_ = 4.0 nm, both the free and reference layers display sharp switching and the as-deposited GMR signal is as high as 6.0 %. As we discussed before, the switching field of the composite reference stack does increase greatly with the Tb_20_Fe_80_ layer thickness. However, the GMR signal decreases as *t*_1_ increased up to 12.0 nm. In addition to the current shunting effect caused by the thicker TbFe layer, the gradual magnetization switching of the free layer may also be responsible for the observed GMR reduction. Note that the minimum GMR value for the SV with thick TbFe occurs at non-zero magnetic field, implying the magnetization orientations of the free and reference layers are not collinear at the remanence state. We consider that the presence of thick TbFe decreases the PMA of free layer, giving rise to its magnetization direction deviating away from the perpendicular direction.

**Fig. 4:**
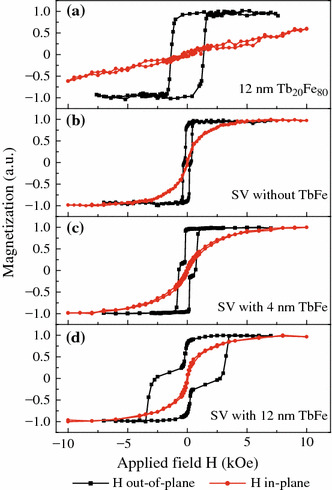
(*Color online*) In-plane and out-of-plane VSM loops measured for pure TbFe of Ta (3.0)/Tb_20_Fe_80_ (12.0)/Ta (3.0) (**a**) and for spin valves of Ta (3.0)/Cu (1.4)/[Ni/Co]_3_/Cu (2.2)/[Co/Ni]_5_/Tb_20_Fe_80_ (*t*_1_)/Ta (3.0), in which *t*_1_ = 0 (**b**), 4.0 nm (**c**), and 12.0 nm (**d**)

In order to understand the TbFe effect on the PMA deterioration of the magnetic free layer, the in-plane and out-of-plane magnetic hysteresis loops were measured by VSM for some representative samples, as shown in Fig. 4. Figure 4a clearly indicates that the easy axis of a pure 12-nm-thick Tb_20_Fe_80_ film is perpendicular to the film plane. Figure 4b–d shows the respective loops for the pseudo SVs with or without a Tb_20_Fe_80_ layer. For the SV without a TbFe layer or with a 4-nm-thin TbFe layer, are both the free and reference stacks display PMA. However, if the TbFe layer thickness is increased up to 12 nm, as shown in Fig. 4d, an obvious in-plane component with very small coercivity appears in the in-plane hysteresis loop, verifying that thick TbFe can cause degradation of the free layer PMA. It is well-known that the TbFe alloy has a much greater magnetostriction effect than TbCo which could modulate the magnetic anisotropy of magnetic thin films [18–20]. As a result, we attribute the observed interfacial PMA reduction of the free layer to the strong stress induced by TbFe during the magnetic hysteresis loop measurement.

**Fig. 5:**
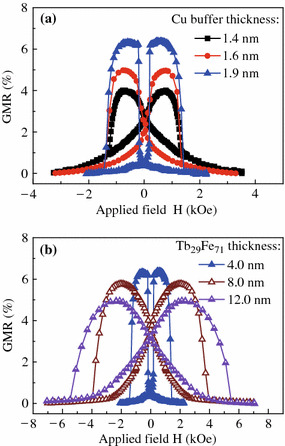
(Color online) The GMR curves for spin valves of **a** Ta (3.0)/Cu (*t*_Cu_ = 1.4, 1.6, or 1.9 nm)/[Ni/Co]_3_/Cu (2.2)/[Co/Ni]_5_/Tb_29_Fe_71_ (4.0)/Ta (3.0) and **b** Ta (3.0)/Cu (1.9)/[Ni/Co]_3_/Cu (2.2)/[Co/Ni]_5_/Tb_29_Fe_71_ (*t*_2_ = 4.0, 8.0, or 12.0 nm)/Ta (3.0)

It was reported that the magnetostriction coefficient increases with the Tb content to Fe ratio within a certain range [20, 21]. In order to further confirm the PMA reduction originating from the TbFe layer, we have fabricated SVs with a Tb-rich Tb_29_Fe_71_ layer. As expected, the Tb_29_Fe_71_ layer has an even serious influence on the PMA strength. Accompanying with a very gradual magnetization reversal process of the free layer, the GMR signal drops significantly down to 4.0 % for the SV with only a 4-nm-thin Tb_29_Fe_71_ layer, shows the solid squares in Fig. 5a. Nevertheless, the undesired easy magnetization slanting behavior can be conquered by increasing the Cu buffer layer thickness *t*_Cu_ owing to the PMA strength enhancement [6]. As shown also in Fig. 5a, by increasing the *t*_Cu_ up to 1.9 nm, the GMR signal recovers to over 6.0 %, and the GMR loop changes from a butterfly shape to a definite square one, revealing that the easy axis of free layer on top of thick Cu lies in the perpendicular-to-plane direction. Unfortunately, as shown in Fig. 5b, if we try to increase the Tb_29_Fe_71_ layer thickness *t*_2_ to 8.0 or 12.0 nm, even 1.9-nm-thick Cu buffer could not be able to keep the free layer magnetization along the perpendicular direction, the GMR curve becomes tilted and the signal decreases again owing to the severe loss of free layer PMA. These results indicate that TbFe is not suitable to be used in the perpendicular SVs to increase the switching field of the reference layer.

## Conclusion

In summary, we have investigated the influence of TbFe alloy on the perpendicular coercivity of exchange-coupled [Co/Ni]_N_/TbFe composite MLs, as well as its role on the GMR signal and free layer easy magnetization orientation for SVs containing a [Co/Ni]_N_/TbFe reference layer. By adjusting the Tb content, TbFe layer thickness and [Co/Ni] repetition number, the magnetization switching field of the composite structure can be greatly tuned, thereby providing an efficient way to achieve large switching field difference in perpendicular SVs. However, it is found that the presence of TbFe layer can decrease the PMA of the free layer, leading to tilted magnetization and reduced GMR ratio. The cause of this phenomenon is attributed to the giant magnetostriction effect of the TbFe alloy.
